# Hot males: thermal biology of males and females during coercive mating in water striders *Gerris lacustris*

**DOI:** 10.1007/s00114-024-01937-1

**Published:** 2024-09-26

**Authors:** Vinicius Marques Lopez, Rhainer Guillermo-Ferreira, Lucia Seip, Stanislav Gorb

**Affiliations:** 1 Lestes Lab. Federal University of Triângulo Mineiro, Uberaba, MG Brazil; 2 Department of Functional Morphology and Biomechanics, Zoological Institute, Kiel University, Am Botanischen Garten 1-9, 24098 Kiel, Germany

**Keywords:** Sexual selection, Thermal biology, Physiology, Insect, Hemiptera

## Abstract

Sexual conflict theory predicts that males that adopt coercive mating strategies impose costs to females during copulation. Nevertheless, conflicting mating strategies may also affect males, although such effects on males are often neglected in the literature. Here, we seek to understand whether male water striders (*Gerris lacustris*) experience higher body temperatures than females during coercive mating behavior. We we explored whether the water temperature affected male and female body temperature differently, considering that water contact by females might serve as a thermal regulator. We built generalized linear mixed models considering the male and female temperature as the dependent variables. Air temperature (as a proxy for solar radiation), water temperature, and sex were used as predictor variables. Our results suggest that males are warmer than females, and despite females coming into contact with water during skimming, this contact does not significantly contribute to lowering their body temperature or improving thermoregulation under the observed conditions. These findings provide novel insights into the thermal biology of water striders. Future studies should focus on addressing whether warmer temperatures confer some advantages to males, such as increased mobility and better ability to hold onto females or impose physiological constraints and fitness costs.

## Introduction

Sexual conflict theory is a well-established concept in the field of evolutionary biology, which predicts that males, who adopt coercive mating strategies, impose costs on females (Clutton-Brock and Parker 1995). For instance, female spiders (*Thanatus fabricii*, Philodromidae) are bitten and immobilized by males as a coercive mating strategy (Sentenská et al. 2020). Even after copulation, females remain more immobile than before, incurring nutritional and foraging costs due to reduced prey capture (Sentenská et al. 2020). Sexual coercion during mating can take place through three main methods: forced copulation, harassment, and intimidation (Clutton-Brock and Parker 1995). Males typically employ physiological, physical, or behavioral tactics to override the female’s choice, with the aim of increasing their chances of reproducing successfully.

In insects, temperature may play a fundamental role not only in behavioral aspects but also in the organism’s physiology and metabolism (Lopez et al. 2019, 2021). However, very few studies have investigated the relationship between temperature and sexual selection, and most of these studies focus on abrupt temperature changes, neglecting the thermal environment which organisms typically inhabit (García‐Roa et al. 2020). It is still scant on the literature whether there is support for hypotheses that address the thermal costs (e.g., females may become immobile due to cold, or males may become too hot because of heat) or benefits (e.g., males may transfer heat to females, and both may become more agile and responsive to mating and against potential predators) of such mating strategies. Experimental evidence is needed to support or refute these hypotheses and make reliable conclusions.

Here, we studied *Gerris lacustris* (Hemiptera: Gerridae), a water strider commonly found near freshwater bodies across Europe. These insects are capable of walking and jumping on the water surface with ease due to their superhydrophobic hairs covering their body and legs (Ma et al. 2020; Meshkani et al. 2023). Mating behavior of water striders is characterized by males attempting coercion through forcible mounting, resulting in vigorous premating struggles with resistant females (Arnqvist 1997; Jabloński and Vepsäläinen 1995). In their natural habitat, these forceful mountings often occur in areas with a high level of sunlight exposure (e.g., Pfenning and Poethke 2006). Thus, we explore whether the male position during coercive mating may results in higher body temperatures for males compared to females. Furthermore, we explored whether the water temperature affected the insects’ body temperature, considering that water might serve as a thermal regulator even without direct contact. We predicted the following: (1) the contact or proximity to water may increase heat loss, so we would expect females to be cooler; (2) males are more exposed to solar radiation, so we would expect males to be warmer; and (3) in both sexes, the solar radiation exposure will have more influence on heat gain.

## Materials and methods

### Data collection

**Fig. 1:**
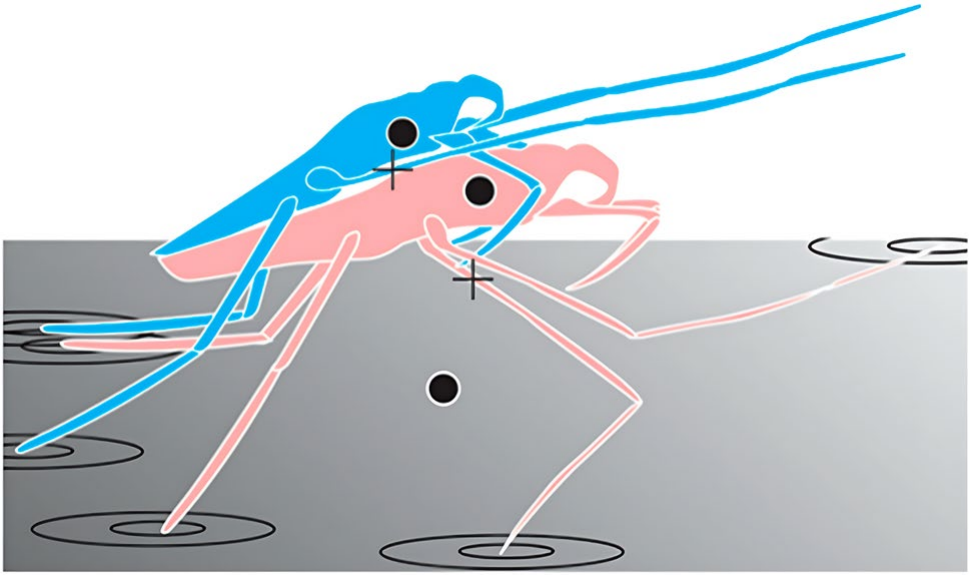
Mating posture in *Gerris lacustris*. Blue, male. Pink, female. Black dots, areas, where average temperature was measured. Black crosses, points, where midleg temperature was measured

**Fig. 2:**
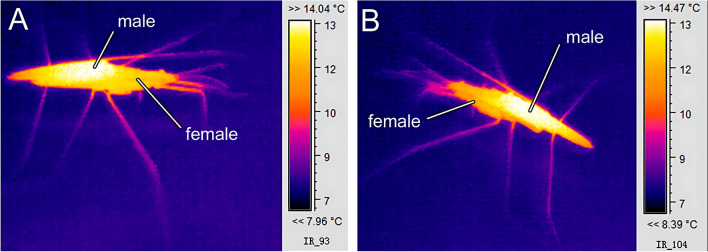
Thermal images of male–female interaction of *Gerris lacustris* during copulation

We conducted data collections at a few ponds in the vicinity of Kiel (Germany), on two occasions. In total, there were 18 days of collection throughout April, September, and October of 2022. In April (i.e., first data collection), we captured thermal images of males and females in mating posture and selected two points with a diameter of approximately 1 mm from each image, focusing on the thorax and midleg central areas (Fig. 1). In September and October (second data collection), we acquired images of separate males and females to obtain the average temperature for both sexes and selected a single point on the thorax for analysis. Temperature data of individual pixels within these areas were averaged and we presented the data in the form of the average temperature within this region (Fig. 2). Finally, the temperature of water and air was also estimated by selecting points close to the specimens.

To analyze the water strider’s thermal behavior, we utilized an IR Trotec IC 080 V camera (Heinsberg, Germany) coupled with a macro lens. All obtained images were later processed and analyzed using the IC-Report DuoVision 1.08.14S software (Trotec, Heinsberg, Germany).

### Statistical analyses

To answer the first two questions, i.e., the contact or proximity to water increases heat loss and males more exposed to solar radiation are warmer, we built generalized linear mixed models (GLMM) with Gaussian distribution, considering the temperature for both sexes (i.e., thorax and midleg temperature for both sex) as a dependent variable. We also considered the relative temperature as a dependent variable, which is calculated by dividing the temperature of the males and females by the water temperature. The use of relative thorax temperature is intended to normalize individual temperatures in relation to the aquatic environment where they reside. While males do not have direct contact with the water to the same extent as females during copulation, the water temperature remains an important reference for the microclimate in which both sexes function. Additionally, the temperature of air (here, a proxy of solar radiation), water, and sex were considered predictor variables. We also considered the collection day as a random factorial variable in all models to account for any variability related to different days of data collection. Hence, we built six different models to address:

- Model 1: What is the effect of air temperature and water temperature on the thorax temperature of males and females?

For this, we used the thorax temperature (i.e., male and female) as a dependent variable and air and water temperature and sex as predictor variables.

- Model 2: What is the effect of air temperature and water temperature on the midleg temperature of males and females?

For this, we used the midleg temperature as a dependent variable and air and water temperature and sex as predictor variables.

- Model 3: How does air temperature influence the relative thorax temperature (thorax temperature divided by water temperature) of males and females?

For this, we used the relative thorax temperature (i.e., thorax temperature divided by water temperature) as a dependent variable and air temperature and sex as predictor variables.

- Model 4: How does air temperature influence the relative midleg temperature (midleg temperature divided by water temperature) of males and females?

We used the relative midleg temperature as a dependent variable and air temperature and sex as predictor variables.

- Model 5: What is the impact of air temperature on the relative difference in thorax temperature between males and females?

In this case, we used the difference in thorax temperature (i.e., male temperature divided by female temperature) as a dependent variable. We considered air temperature and sex as predictor variables.

- Model 6: What is the impact of air temperature on the relative difference in midleg temperature between males and females?

We used the difference in midleg temperature (i.e., male temperature divided by female temperature) as a dependent variable. We considered air temperature and sex as predictor variables. For analyses, we used the *glmmTMB* package (Magnusson et al. 2017) to estimate the GLMM results, and we generated the plots using the *ggplot2* package (Wickham and Chang^2016^).

For our third question i.e., in both sexes, the solar radiation exposure will have more influence on heat gain, we employed linear regression analysis. All analyses were performed using the R environment (R Core Team, 2022) and all data are available in the Zenodo repository (see in 10.5281/zenodo.7893385). Here, “proximity to water” refers to considering the water temperature in the environment surrounding the insects, rather than a direct measurement of the distance of individuals to the water surface. For our study, we defined thermoregulation as the ability of water striders to sustain or achieve a body temperature that supports their physiological functions, considering their exposure to solar radiation and water temperature.

## Results

A total of 87 thermal images of males and females in mating posture were recorded in the first field collection and nine images were recorded in the second field collection.

### Influence of water on body temperature

**Fig. 3:**
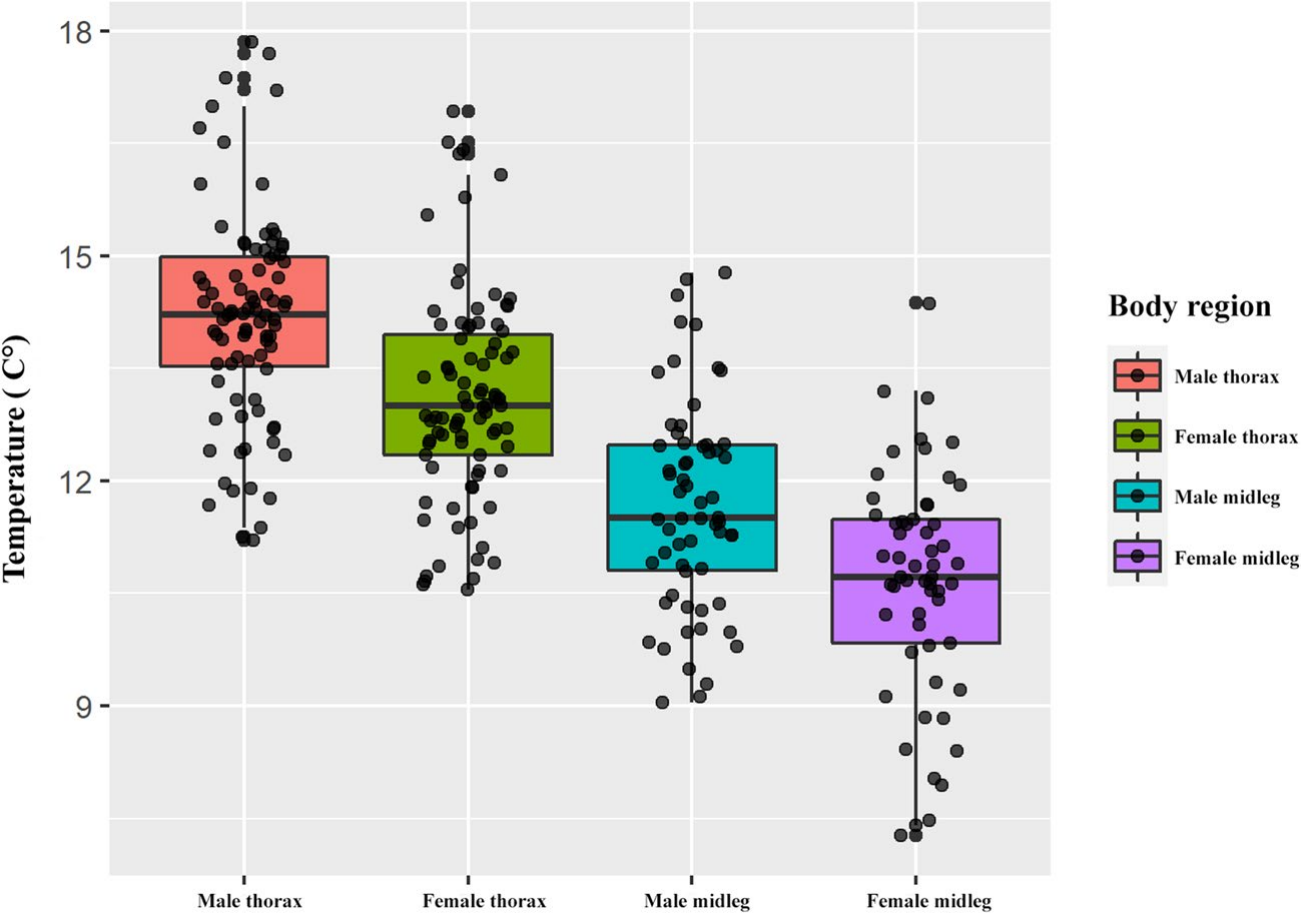
Boxplot of body temperatures by body regions in water strider *Gerris lacustris*

**Fig. 4:**
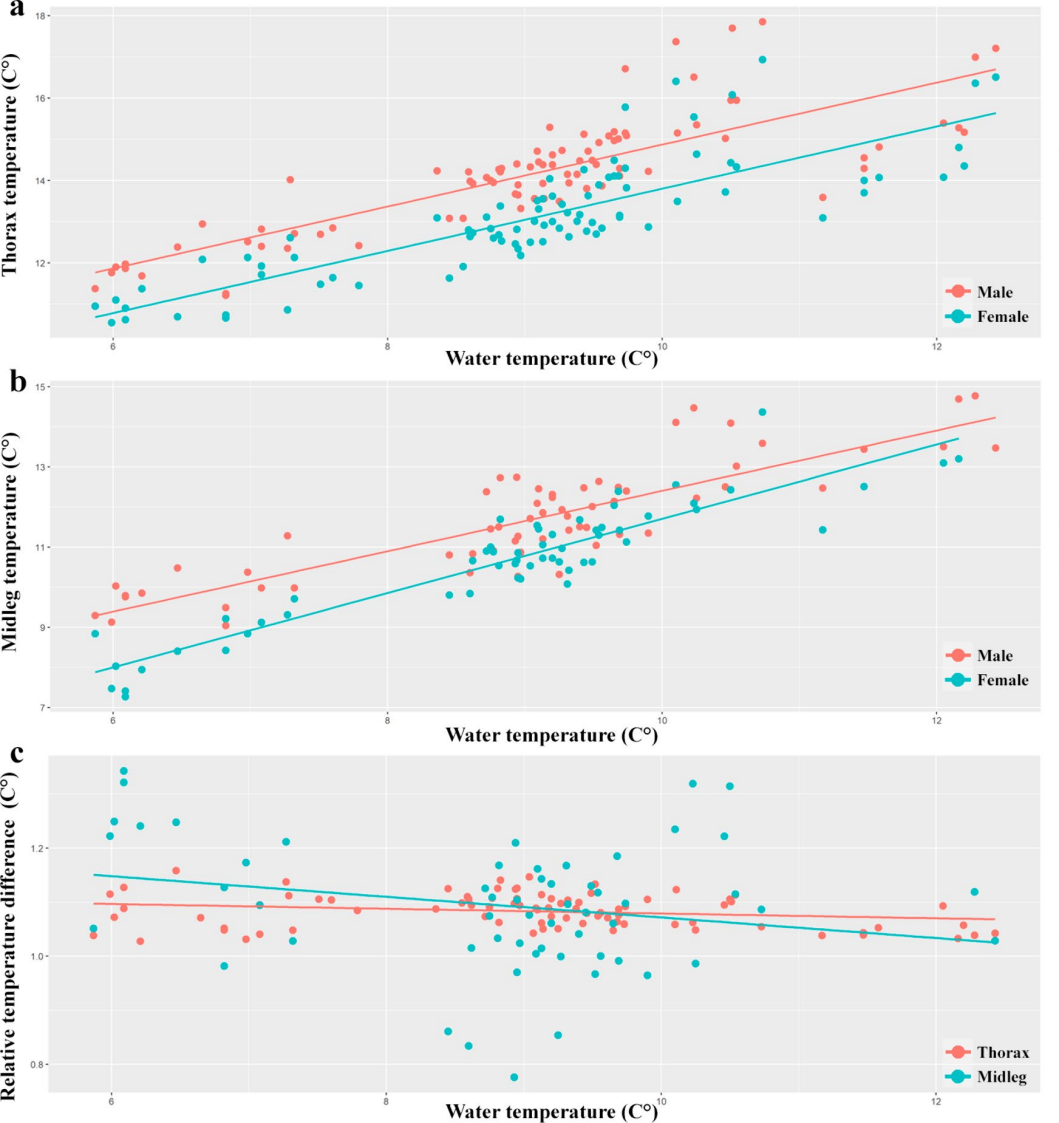
Temperature in copulating male and female water strider *Gerris lacustris*. **A** The relationship between water temperature and thorax. **B** The relationship between water temperature and midleg. **C** The relative temperature difference of males to females (male’s temperature divided by female’s temperature). The results show that males are warmer than females

Our initial hypothesis proposed that proximity to water might enhance heat dissipation. However, our generalized linear mixed models (GLMM) analysis shows that water temperature did not significantly impact the thorax or midleg temperatures of either males or females. Instead, our analysis indicates that air temperature plays a significant role in determining the body temperature of water striders, particularly affecting the temperature differences between males and females (see Models 1 and 2, Table 1, Figs. 3 and 4). Our models do not support the notion that contact with water facilitates heat loss (Models 3, 4, 5, and 6).

**Table 1:** Summary models to explain the thorax and midleg temperature in water striders. Significant results in bold for GLMM models with Bonferroni correction applied (*α* = 0.003)

Predictor variables	Estimate	std. error	*z* value	*p*
Model 1—Male–female thorax temperature				
Sex	− 1.07356	0.11888	− 9.031	** < 0.001**
Water temp	0.67699	0.04914	13.778	** < 0.001**
Air temp	− 0.15826	0.06158	− 2.570	0.01
Model 2—Male–female midleg temperature				
Sex	− 0.88173	0.12042	− 7.322	** < 0.001**
Water temp	0.81680	0.04778	17.094	** < 0.001**
Air temp	− 0.03766	0.07167	− 0.525	0.599
Model 3—Relative male–female thorax temperature				
Sex	− 0.12158	0.01657	− 7.337	** < 0.001**
Air temp	0.06403	0.04314	1.484	0.137
Model 4—Relative male–female midleg temperature				
Sex	− 0.10551	0.01554	− 6.789	** < 0.001**
Air temp	0.04764	0.02978	1.600	0.109
Model 5—Relative male–female thorax temperature differences				
Water temp	0.00118	0.00242	0.486	0.6267
Air temp	0.01153	0.00304	3.796	** < 0.001**
Model 6—Relative male–female midleg temperature differences				
Water temp	0.01686	0.02745	0.614	0.539
Air temp	0.04756	0.03524	1.349	0.177

**Fig. 5:**
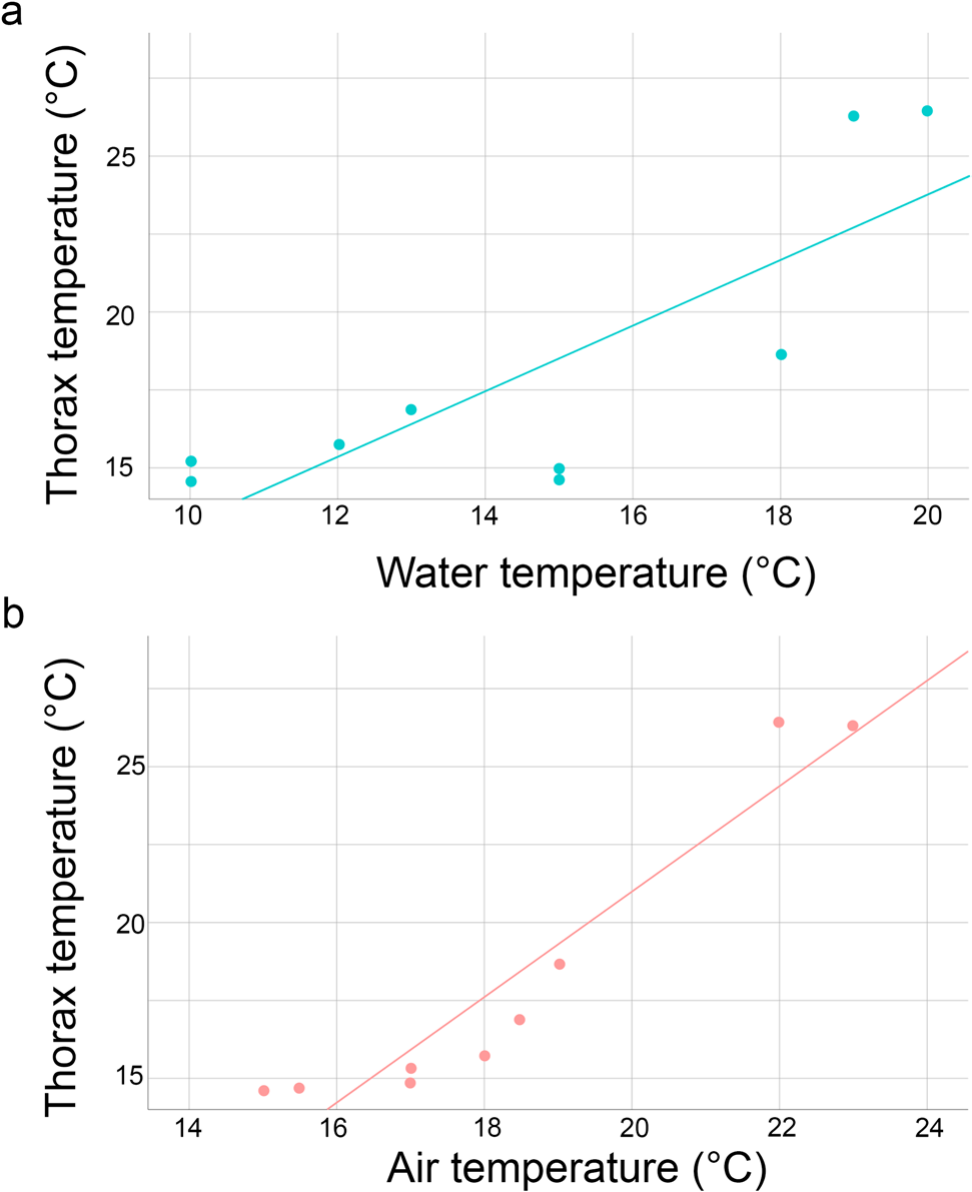
Linear regression model of male water striders (*Gerris lacustris*) temperature based on water (**A**) and air temperature (**B**). The values represent males decoupled of females

We observed a significant difference in thorax temperature between males and females (Model 1, Table 1, Fig. 4A), as well as in midleg temperature (Model 2, Table 1, Fig. 4B). However, this difference in temperature between sexes was not explained by water temperature (Models 5 and 6). Additionally, there was significant variation in relative temperatures (i.e., individual temperature divided by water temperature) between males and females (Models 3 and 4, Table 1). Although water temperature influenced temperature differences when males and females were separated (*y* = 1.0494*x* + 2.7705, *R*^2^ = 0.6624 in Fig. 5A), it was not a primary factor in explaining the temperature differences between sexes.

### Influence of air/radiation on body temperature

Our analysis aimed to determine the possible impact of air temperature, serving as a proxy for solar radiation, on the body temperature of water striders and how these effects differ between males and females. Our results show a positive correlation between the difference between male and female temperature and air temperature (Model 6, Table 1). That is, when the air temperature is higher, the difference between male and female temperature is higher. This suggests that higher air temperatures may increase the thermal contrast between sexes. However, the results were not significant for midleg temperature (Model 2) or relative temperatures (Models 3 and 4).

Our results regarding the influence of air temperature (i.e., proxy of solar radiation) exposure on heat gain for males, independent of females suggest a significant impact on the temperature of water striders (as represented by the linear regression equation *y* = 1.6948*x* − 12.908 and *R*^2^ = 0.9023 in Fig. 5B).

## Discussion

Here, we explored the idea that the male position during coercive mating would increase exposure to solar radiation, increasing heat gain. We found that solar radiation (i.e., air temperature) has a significant impact on heat gain, suggesting that the temperature of water striders is largely influenced by air temperature. Our results suggest that the relative and absolute difference in temperature between males and females is mainly due to air temperature. This supports the hypothesis that male water striders may gain more heat than females during mating. However, our results indicate evidence contrary to our prediction that proximity to water would increase heat loss.

Temperature is an important factor in the ecology of ectothermic animals, but it's been overlooked when it comes to sexual selection (García-Roa et al. 2020; Leith et al. 2022). Our results suggest that males of water striders may possibly gain more heat due to their coercive mating behavioral strategy. Eastern mosquitofish males (*Gambusia holbrooki*, Poeciliidae), who also exhibit a coercive strategy, showed reproductive behaviors within a wide temperature range (14 to 38 °C), representing one of the widest reproductively active temperature ranges in ectotherms (Wilson 2005). Male fiddler crabs (*Uca pugilator*, Ocypodidae) accept staying in habitats with high temperatures and food restrictions when the availability of females is high (Allen and Levinton 2014).

The phenology of *G. lacustris* can be strongly influenced by the type of habitat occupied. For instance, characteristics such as life cycle, proportion of long-winged individuals, and egg and larval development rate may be severely altered in species occupying warm open habitats or colder forested habitats (Pfenning and Poethke 2006). Our study results suggest that male *G. lacustris* may need to thermoregulate more often than their female mates. However, an alternative hypothesis that could be explored is that increased temperatures may increase the activity levels of males, which could provide benefits during cooler days or particularly at the start of the mating season in spring.

The thermal plasticity and life history variability of water strider species is relatively well documented. For instance, on a small geographical scale in Germany, populations of *G. lacustris* were more abundant in open habitats compared to forest ponds (Pfenning and Poethke 2006). Studies have also shown that populations of *G. remigis* occupying warm environments can reach nine times higher growth rates compared to cold streams (Fairbairn 1985). Additionally, higher temperatures (30 ± 2 °C) can induce a higher proportion of macropterous adults in *Gerris paludum insularis* (Harada and Taneda 1989). The same way, the thermal tolerance range of *Aquarius paludum* nymphs was observed to be from − 3.38 to 45.1 °C (Harada et al. 2010) Therefore, higher temperatures are not necessarily a problem if the species has thermal adaptability to handle the heat stress imposed by coercive mating behavior.

The results showed evidence that contradicts our prediction that proximity to water would increase heat loss. Specifically, the results are not significant for the relative temperature (*t*_males_/*t*_*f*emales_) in relation to water temperature, which means that water does not affect differences between males and females. The bodies and legs of these insects have a unique superhydrophobic capacity, repelling water and having an air gap even when in contact with water (Ma et al. 2020). This phenomenon is caused by dense bristles that make the legs superhydrophobic (Gao and Jiang 2004). A hypothesis is that this air gap may prevent heat loss to the water. Therefore, we suggest further experiments to analyze whether superhydrophobic structures can obstruct heat loss.

In cases of thermal stress, ectothermic organisms typically address these challenges through behavioral thermoregulation (e.g., moving to microhabitats with more favorable temperatures) (Ma and Ma 2012; Sunday et al. 2014; Bodlah et al. 2023). The evolutionary pressure to manage these thermal costs can drive the development of behavioral and physiological strategies that minimize heat exposure (González‐Tokman et al. 2020; Bodlah et al. 2023). For instance, male water striders may evolve to seek cooler microhabitats or adjust their mating times to avoid peak solar intensity. In this context, natural selection could favor males who balance the trade-off between coercive mating behaviors and thermal stress, potentially refining mating strategies to optimize both reproductive success and thermoregulation. However, other species of Gerridae, such as some oceanic skaters (*Halobates*), appear to exhibit high resistance to elevated temperatures and can adapt to seasonal changes in sea surface temperatures (Harada et al. 2018). Therefore, studies quantifying the thermal tolerance capacities of *G. lacustris* are necessary to understand the evolutionary implications of our findings.

We recognize that while our study demonstrates males exhibit higher body temperatures than females, we have not explicitly linked these thermal differences to physiological costs. It remains unclear whether elevated body temperatures in males directly induce fitness costs or if warmer temperatures might confer certain advantages, such as enhanced mobility or improved grip during mating. This ambiguity limits our ability to draw definitive conclusions about the evolutionary and behavioral implications of our findings.

By examining the thermal environment and the thermal biology of water striders during mating, we can gain a more comprehensive understanding of how temperature may influence mating behaviors. We suggest that new studies should concentrate on the physiological consequences of prolonged exposure to the solar radiation in *Gerris lacustris*. Future research also should aim to investigate whether these higher temperatures are costly or beneficial to male and female mating success, endurance, or other performance metrics. Finally, the length of copulation and postcopulatory guarding and the different sizes of males should also be studied in the context of thermal biology.

## Data Availability

The datasets generated and analyzed during the current study are available in the Zenodo repository (see in 10.5281/zenodo.7893385).
